# Screening and isolation of cold-adapted cellulose degrading bacterium: A candidate for straw degradation and *De novo* genome sequencing analysis

**DOI:** 10.3389/fmicb.2022.1098723

**Published:** 2023-01-13

**Authors:** Zhigang He, Baopeng Ding, Qurban Ali, Huiyu Liu, Ying Zhao, Xiujuan Wang, Yingzuo Han, Huan Dong, Praveen Kumar Divvela, Yinghua Juan

**Affiliations:** ^1^Institute of Plant Nutrition and Environmental Resources, Liaoning Academy of Agricultural Sciences, Shenyang, China; ^2^College of Forestry, Shanxi Agricultural University, Taigu, Shanxi, China; ^3^Key Laboratory of Integrated Management of Crop Diseases and Pests, Ministry of Education, Department of Plant Pathology, College of Plant Protection, Nanjing Agricultural University, Nanjing, China; ^4^General Manager, Contec Global Agro Ltd., Abuja, Nigeria

**Keywords:** low temperature, Comamonas sp. K1, corn straws, genome analysis, novel bacteria

## Abstract

Degradation of crop straw in natural environment has been a bottleneck. There has been a recent increase in the exploration of cold-adapted microorganisms as they can solve the problem of corn straw degradation under low temperatures and offer new alternatives for the sustainable development of agriculture. The study was conducted in low-temperature (10°C) and high-efficiency cellulose-degrading bacteria were screened using carboxymethyl cellulose (CMC) selection medium and subjected to genome sequencing by the third-generation Pacbio Sequl and the second-generation Illumina Novaseq platform, and their cellulase activity was detected by 3,5-dinitrosalicylic acid (DNS) method. The results showed that the low-temperature (10°C) and high-efficiency cellulose-degrading bacterium *Bacillus subtilis* K1 was 4,060,823 bp in genome size, containing 4,213 genes, with 3,665, 3,656, 2,755, 3,240, 1,261, 3,336 and 4,003 genes annotated in the non-redundant protein sequence database (NR), Pfam, clusters of orthologous groups of proteins (COGs), Genome Ontology (GO), Kyoto Encyclopedia of Genes and Genomes (KEGG), and Annotation databases, respectively. In addition, a large number of lignocellulose degradation-related genes were annotated in the genome. The cellulose activity of *B. subtilis* K1 was higher, exhibiting the highest activity of endo-β-glucanase (24.69 U/ml), exo-β-glucanase (1.72 U/ml) and β-glucosaccharase (1.14 U/ml). It was found that through adding cold-adapted cellulose-degrading bacteriaK1 in the corn straw composting under 6°C (ambient temperature), the average temperature of straw composting was 58.7°C, and higher 86.7% as compared to control. The HA/FA was higher 94.02% than the control and the lignocellulose degradation rate was lower 18.01–41.39% than the control. The results provide a theoretical basis for clarifying the degradation potential of cold-adapted cellulose-degrading bacteria and improving the cellulose degradation efficiency.

## 1. Introduction

Northeastern part of China is the major grain producing region in China, contributing to 25% grain production and 33% grain transfer amount, which serves as a “stabilizer” and “ballast” ensuring the national food security (Cui et al., 2016; Liu et al. 2017). The production of crop straws has emerged owing to the rapid development of agriculture with annual output of about 865 million tons, and a resource utilization rate of 81.68% (Zhang et al., 2021). Improper treatment of straws leads to serious environmental pollution. Therefore, reasonable treatment of crop straws is of great significance for developing green agriculture, protecting local environment, increasing the resource utilization rate, improving the soil matrix, and raising the agricultural development quality (Liu et al., 2021).

Crop straws degrade slowly under natural conditions in Northeastern part of China due to the limitation of the overall economic conditions and regional climate characteristics (Yao et al., 2009). At present, the crop straws are treated in a rough way, mostly by burning, which is not conducive to environmental protection, agricultural sustainable development, and virtuous cycle of cultivated land (Shi et al., 2019). However, the degradation of crop straws in the natural environment is faced with many problems (Liu et al., 2017). First, the straw is primarily composed of lignin, cellulose, and hemicellulose, all of which are aromatic polymer compounds with a complex 3D structure difficult to degrade in the natural environment (Muhammad et al., 2017). Second, the natural degradation rate of straws further decreases due to the lower mean temperature and frequent soil freezing and thawing (Mu et al., 2013). Therefore, it is urgently needed to develop efficient, feasible, green, and energy-saving straw treatment methods under low temperature.

As extensively reported in literature, the synergistic action of multiple carbohydrate-active enzymes (CAZymes) is required for the degradation of straw cellulose polymers, and these enzyme families degrade carbohydrates through multiple complementation and coordination of oxidative, hydrolytic, and non-hydrolytic activities (Pollegioni et al., 2015). Three enzymes, i.e., endoglucanase, exoglucanase, and β-glucosidase (BG) are involved in the hydrolysis of cellulose into glucose monomer (Horn et al., 2012; Ma et al., 2016). Cellulose-degrading enzymes secreted by fungi, such as *Trichoderma* (Karlsson et al., 2002), *Aspergillus* (Hasper et al., 2000) and *Penicillium* have been widely studied in microorganisms (Xu et al., 2007; Qiao et al., 2013; Du et al., 2018). In contrast to fungi, bacteria characterized by a higher growth rate, expression of multi-enzyme complexes, and tolerance to extreme environments have become the emphasis of cellulose production research (Chuanjiao et al., 2021). Currently, many kinds of bacteria have been reported to be able to degrade lignin, for example, *Actinomycetes*, *Streptomyces*, *Arthrobacter*, *Micromonospora* and *Nocardia*, possess strong degradation ability. *Acinetobacter*, *Flavobacterium*, *Micrococcus*, *Pseudomonas* and *Xanthomonas* are the non-filamentous bacteria capable of degrading lignin. At the same time, the biological versatility of bacteria with high genetic variability can be enhanced through genetic evolution (Chen, 2013). In addition, bacteria have a wide adaptation range of temperature and pH, so lignocellulose-degrading bacteria play a more important role under low temperature. In recent years, there has been a great interest in discovering new enzymes from extremophilic microorganisms, for industrial purposes. With scholars’ increasing attention to microbe resources, cryogenic cellulase-producing bacteria have been excavated in different cold habitats, including perennial glaciers, permafrost, seabed silt, basins, and sediments. Some researchers found that *Sphingobacteria, Flavobacteria,* and *Bacillus,* in different Antarctic environments, have the ability to adapt to cold temperatures and have the ability to degrade arabinose and hemicellulose (Smith et al., 2018; Cho et al., 2021). Among them, *Bacillus subtilis* can form highly resistant spores to various extreme environments, with strong environmental adaptability (Kattia, 2021).

In this study, the bacteria with strong cellulose-degrading ability were screened and isolated using the Congo red carboxymethyl cellulose (CMC) medium, and the genetic basis of their cellulose-degrading ability was revealed through whole genome sequencing and analysis, and their ability of cellulose degradation was measured by means of cellulose activity assay. Besides, new cold-adapted cellulose-degrading strains were prepared based on low-temperature cellulose-degrading strains obtained by previous enrichment culture, and their straw-degrading effect was further validated *via* winter corn straw composting experiment, which could provide a theoretical basis for comprehensive utilization of straws.

## 2. Materials and methods

### 2.1. Enrichment isolation and screening of strains

The appropriate amount of winter forest soil samples from northeast China were collected in natural environments below 10°C, loaded in sterile self-sealing bags, brought back to the laboratory, and refrigerated at 4°C. 10.0 g of the above samples were placed in a triangular bottle with 90 ml of sterile water for 20 min, followed by fully absorbing 1 ml of supernatant into 100 ml of enrichment medium at 10°C, observation, and shaking once a day. When the straw section became loose and soft and the culture medium became darker, it was transferred to the fresh enrichment medium with 5% inoculum and then repeated four times.

### 2.2. Medium formulation and cellulose activity assay

Hutchinson enrichment liquid medium containing: KH_2_PO_4_ 1 g/l,NaCl 0.1 g/l,NaNO_3_ 2.5 g/l, MgSO_4_·7H_2_O 0.3 g/l, FeCl_3_·6H_2_O 0.01 g/l, CaCl_2_ ·6H_2_O 0.01 g/l, and corn straw 5 g/l, pH = 7.0 and the Luria-Bertani (LB) liquid and solid media containing: tryptone 10 g/l, yeast extract 5 g/l, and NaCl 10 g/l, pH = 7.0; Carboxymethylcellulose sodium culture (CMC) medium: sodium CMC 5 g/l, NaNO_3_ 3 g/l, KH_2_PO_4_ 1 g/l, MgSO_4_·7H_2_O 0.5 g/l, KCl 0.5 g/l, FeSO_4_ 0.01 g/l, and agar 15 g/l, pH = 7.0 were prepared and used in this study. The activities of different cellulases in the bacterial solution were detected by 3,5-dinitrosalicylic acid (DNS) method (Lu et al., 2015). Briefly, the degradation ability was detected using cellulose substances CMC (Somogyi, 1926), absorbent cotton, and salicin as substrates, in which CMC was the substrate for endo-1,4-β-glucanase (CMCase), absorbent cotton was the substrate for exo-1,4-β-glucanase (C_1_), and salicin was the substrate for BG (Somogyi, 1952).

### 2.3. Isolation and screening of strains

The enriched bacterial solutions with dilution ratios of 10^−4^, 10^−5^, and 10^−6^ were coated on the above isolation medium, and cultured at 10°C for 3 d to obtain pure culture. Then the purified strains were inoculated into the cellulose Congo red medium and cultured at 10°C for 4 d, followed by preliminary screening based on the ratio of the diameter of the hydrolysis circle (D) to the diameter of the colony (d; D/d). Finally, 1 ml of the suspension (1 × 10^9^ bacteria) of the cellulose-degrading strains screened was inoculated into the liquid fermentation medium (50 ml in a 250 ml conical flask) and cultured by constant temperature shaking at 10°C and 160 r/min for 6 d, and its enzyme activity was measured (Liu et al., 2014).

### 2.4. Molecular identification of bacteria

Polymerase chain reaction (PCR) amplification was conducted with 16S ribosomal ribonucleic acid (rRNA) V3-V4 as the amplification region, and 27F (5’-AGAGTTTGATCMTGGCTCAG-3′) and 1492R (5’-TACGGYTACCTTGTACGACTT-3′) were used as forward and reverse primers. Amplicons were examined by 1% agarose gel electrophoresis (Chuanjiao et al., 2021). A phylogenetic tree of the sequences was retrieved from National Center for Biotechnology Information (NCBI) GenBank and the reference sequences were constructed by the neighbor-joining method using MEGA 7. The PCR was conducted with following conditions: pre-denaturation at 94°C for 2 min, 30 cycles of denaturation at 94°C for 30 s, annealing at 55°C for 30 s and extension at 72°C for 100 s, and repair and extension at 72°C for 2 min.

### 2.5. Genomic DNA extraction and sequencing

A single colony was inoculated into the LB liquid medium, cultured in a shaker at 30°C and 170 r/min for 2 d, and collected by centrifugation at 8,000 rpm for 3 min. The genomic DNA was extracted from the strains through CTAB method (Motham et al., 2014), and the total microbial DNA was extracted using Power Soil^®^ DNA Isolation Kit. The purity and concentration of the total DNA were measured by virtue of a NanoDrop ND-1000 nucleic acid quantitation instrument. The purified DNA was sent to Wuhan OneMore Technology Co., Ltd. for whole genome sequencing. The genome was filtered and assembled by HGAP 2.0 (Westbrook et al., 2015).

### 2.6. Gene prediction and functional annotation

The cultured bacteria were collected at the initial logarithmic stage and were sent to Wuhan Guao Technology Co Ltd. for genome extraction and sequencing. Illumina PCR adapter reads and low-quality reads from the paired-end were filtered using the readfq (Version 10) remove reads with less than a certain percentage of low-quality bases (mass value B 38 or default is 40 bp), a certain percentage of reads with N bases (default is 10 bp), overlap exceeds a certain threshold (default is 15 bp) and the possibility reads originating from the host (Chuanjiao et al., 2021). Coding regions of coding genes were predicted by Glimmer 3.0. The predicted genes were aligned to NCBI non-redundant protein sequence database (NR), clusters of orthologous groups of proteins (COGs) database, Swiss-Pro, Genome Ontology (GO) and Kyoto Encyclopedia of Genes and Genomes (KEGG) database by BLAST for gene functional annotation.

### 2.7. Solid straw composting fermentation

*Bacillus subtilis* K1 and the pre-screened composite strains were selected for subsequent straw composting experiment. Corn straws were the composting materials, containing 11.9% water, 0.62% total N, 0.09% total P, 0.28% total K, C/N 37.42, and urea (46% N). Three treatments were set up in the composting experiment:

Bio-composting (M): About 1,000 kg of corn straws were composted and added with 5 l (5 × 10^7^ CFU/l) of composite strain (included yeast, and *Trichoderma viride*, obtained from The Liaoning Academy of Agricultural Sciences Institute of Plant Nutrition and Environmental Resources Microbiology Laboratory) and 10 kg of urea. (2) Bio-composting (MC): About 1,000 kg of corn straws were composted, with 2.5 l each of *Bacillus subtilis* K1 and composite strain fermentation broth and 10 kg of urea. (3) Control (CK): About 1,000 kg of corn straws were composted without adding microbial agents but added with 10 kg of urea. The experiment was repeated 3 times.

Specifically, a flat head land was selected and stacked with crushed corn straws, and a little microbial agents and urea were sprinkled on the surface for every 20 cm rise. Meanwhile, the straws were compacted by treading. The composting height was kept at 1.5–1.8 m, and the outer surface was covered with an agricultural plastic film. The experiment was from March 13, 2019, and the composting lasted for 45 d in total. Test site: Shenyang Xidi Testing Base (123°55′43.16″ E, 41°82′17.74” N).

### 2.8. Physiochemical properties

During the composting process, temperature changes were detected. At the same time, the content of N, P_2_O_5_, K_2_O and active organic matters and the pH of straws were measured in accordance with China’s organic fertilizer standard (NY 525–2012). The humus components were determined according to Liu Xiaojing’s method Sodium pyrophosphate-sodium hydroxide extraction, potassium dichromate oxidation capacity method (Liu et al., 2017). The content of cellulose and hemicellulose was measured through Van Soest method (Zhang et al., 2017). The morphological and physicochemical indexes of the strains were observed, and the OD_600_ of the strains under different carbon sources, pH, and temperature conditions were determined (Liu et al., 2017) with some modifications.

### 2.9. Data analysis

Every experiment was conducted using a completely random design. The results of three replicates (*n* = 3) were expressed as SD (standard deviations). Following an Analysis of variance (ANOVA), the means were determined using Tukey’s Honestly Significant Difference (HSD) test at *p ≤* 0.05. The data were statistically analyzed using IBM SPSS Statistics 22.0. Origin’s graphics and analysis software was used to create the graphical representations (Version 2022, OriginLab Corporation, Northamptom, MA, United States).

## 3. Results

### 3.1. Isolation and identification of cellulose-degrading bacteria

**Figure 1:**
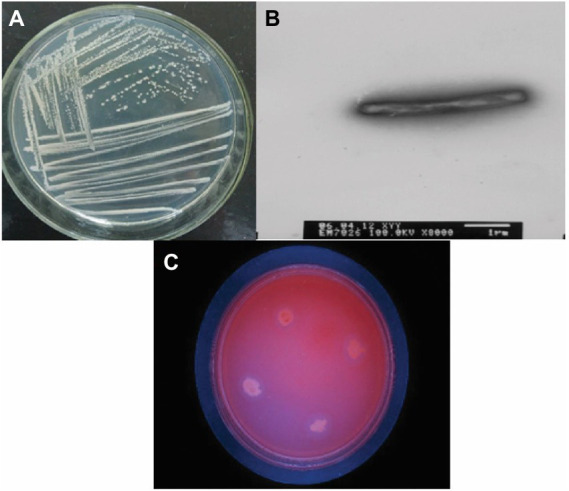
**(A)** Plate photo of the *Bacillus subtilis* K1. **(B)** Scanning electronic microscopy (SEM) photo of the *Bacillus subtilis* K1. **(C)** CMC sodium culture plate photo of the *Bacillus subtilis* K1.

**Figure 2:**
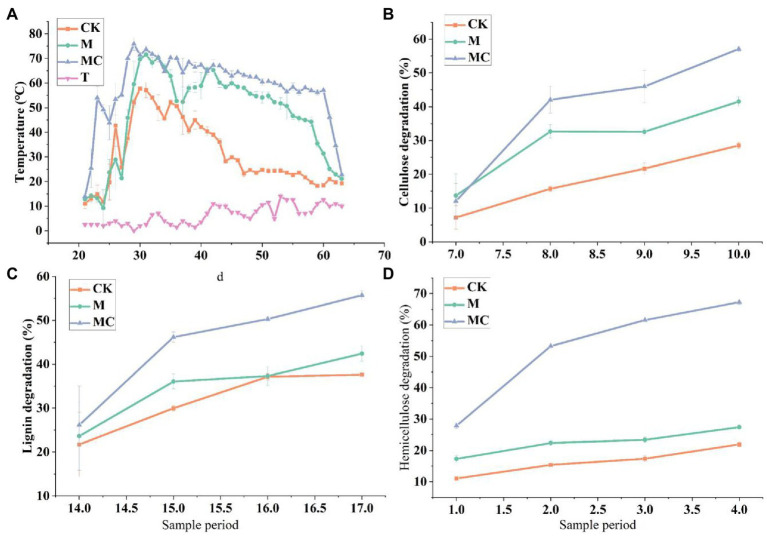
Changes of nutrient mixtures in the process of corn straw producing compost **(A)**. Changes of humus mixtures in the process of corn straw producing compost **(B)**. Vertical bars on graphs indicate the standard deviation of the mean (*n* = 3). The small letters show significant differences between the samples. Tuckey’s HSD test was used to recognize a significant difference at *p ≤* 0.05 between the treatments.

**Figure 3:**
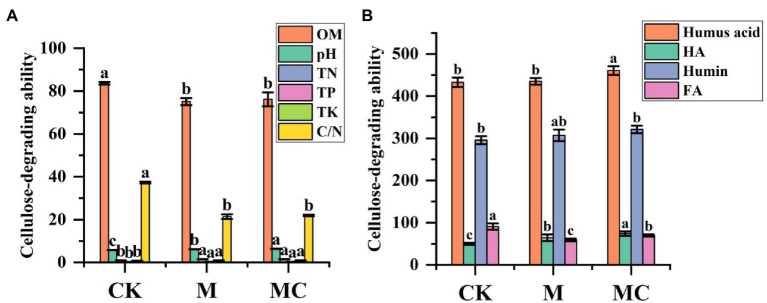
The 30 cellulose decomposing microorganism D/d **(A)**. Accurate measurement of cellulose activities in cellulose decomposing microorganisms **(B)**. Vertical bars on graphs indicate the standard deviation of the mean (*n* = 3). Tuckey’s HSD test was used to recognize a significant difference at *p* ≤ 0.05 between the treatments.

The bacteria were cultured using CMC selection medium, and the isolated strains were subjected to the Congo red plate test, forming transparent circles as shown in (Figure 1). Therefore, K1 was identified as *B. subtilis* and named *B. subtilis* K1. The *B. subtilis* K1 is a regular single cell, 2–5.5 μm long and ~ 0.3–0.4 μm wide, with flagellum around its body as shown in its SEM micrograph (Figure 1B), and it is a small, round, moist, smooth, viscous, and easy to pick (Figure 1A). It has G+ rod, milk-white round colony with the growing conditions of 5–40°C (optimal temperature 25°C), pH 3.3–8.5 (optimal pH 6.5), optimal carbon source is sodium CMC. It entered the logarithmic period at 8 h, with the highest OD_600_ value at 30 h, and then entered the stabilization period (Figure 2A). The ratio of the diameter of the transparent circle to the diameter of the colony (D/d) was calculated to preliminarily determine the strains with strong cellulose-degrading ability. As shown in Figure 3B, the mean D/d was the highest in K, followed by W, V, and Z. A total of 12 strains with larger transparent circles were screened for further screening. The K1 was higher, among which CMCase had the highest activity (24.69 U/ml), followed by endoglucanase (1.72 U/ml) and exoglycanase (1.14 U/ml). Considering the growth rate and the cellulose-degrading ability, K1 was further studied (Figure 3A).

### 3.2. Changes in physicochemical properties of straw composting

#### 3.2.1. Temperature

**Figure 4:**
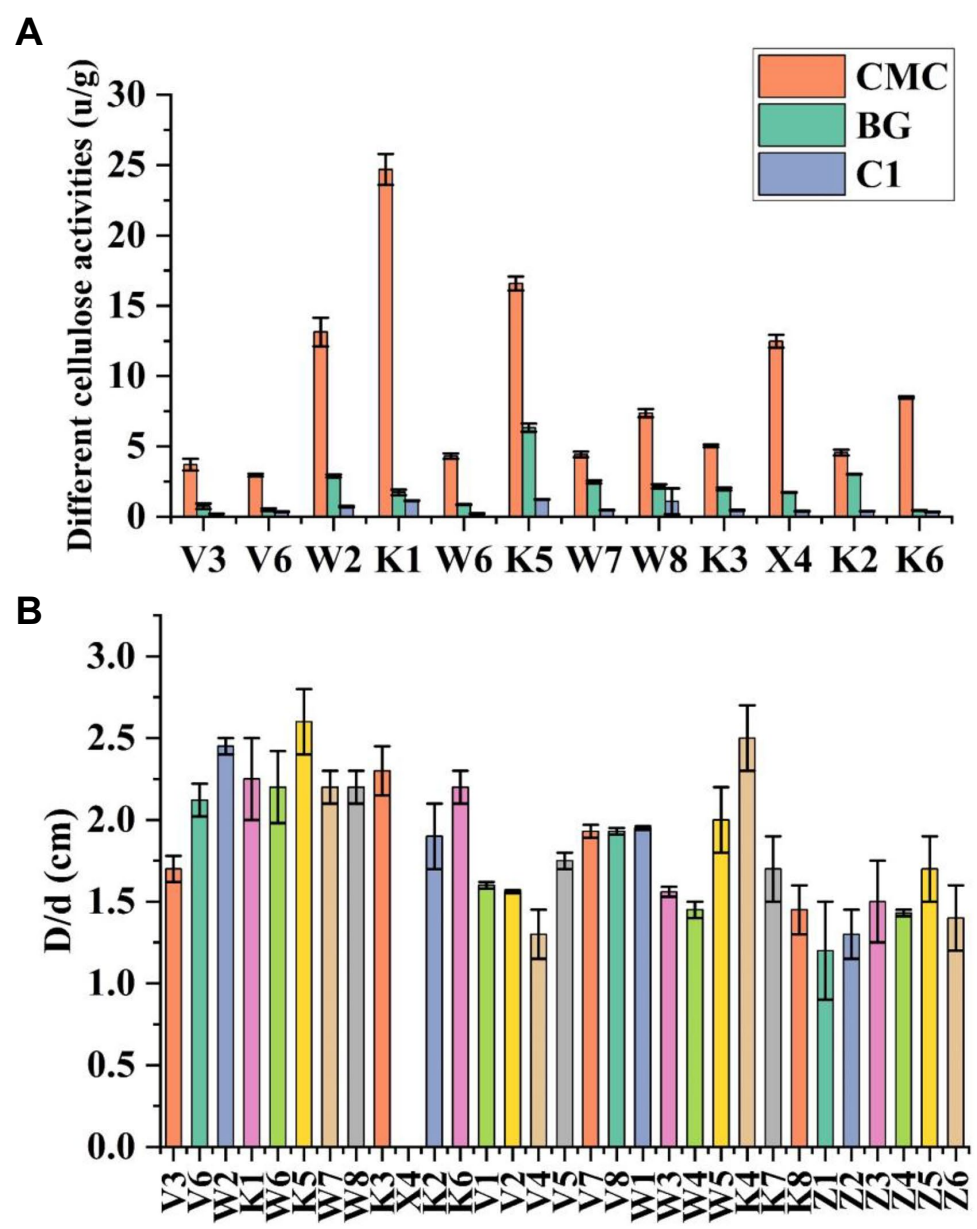
**(A)** Changes in the temperature of mixtures of corn straw producing compost. Changes of Cellulose mixtures in the process of corn straw producing compost. **(B)** Hemicellulose degradability. **(C)** cellulose degradability. **(D)** Lignin degradation. The graphical representation of the degradation changes of corn straw at different time intervals.

Temperature is the most significant factor affecting microbial activity, and it is decisive for the composting reaction rate, which often serves as a macro indicator for microbial biochemical activity in composting. Both the excessively low and high temperatures affect the composting reaction process. In this experiment, the initial ambient temperature of composting was set at 2°C, and the temperature for both M and MC increased from low to high and then gradually decreased, complying with the change rule of composting temperature. In the early stage of composting, the microbes massively multiplied, rapidly decomposed, and utilized simple organic matters in the material to generate biological heat, so the composting temperature rose (50.8°C at 3 d and 53.4°C at 9 d) and entered a high temperature period, reaching the peaks of 68.4°C and 74.8°C, respectively. Moreover, the temperature maintained at above 50°C for 16 d. In MC, the temperature rose rapidly, exceeded 50°C at 3 d, reached the peak (above 70°C) at 10 d and then started to decline, the high temperature above 60°C lasted for 31 d, and then the temperature dropped below 30°C. In M, it took a longer time to enter a high temperature period since long-term normal temperature microbial fermentation agents were used. In CK, the temperature rise during accumulation was slower than that in MC (significantly lower than the highest temperature in MC), and no obvious high temperature period was found, with greater temperature fluctuations. According to the statistical analysis of the grouped data, the temperature had a highly significant difference between MC and M during the whole fermentation process. As observed in Figure 4A, under an average outside temperature of 6°C, the average temperature of straw composting (58.7°C) was 86.7% higher than that of CK after the low-temperature corn straw decomposing strains were added. It can be seen that the low-temperature composite strain had a significant temperature-rise effect on low-temperature composting fermentation, and it quickly raised the temperature and ferment under a low outside temperature, thus achieving rapid decomposition.

#### 3.2.2. Decomposition of hemicellulose, and lignocellulose

As shown in Figure 4B, the lignocellulose and hemicellulose exhibited significant differences between MC and CK after 1-month composting. The indicators significantly declined in MC. The reason could be that the low-temperature composite strain not only degrades the easily decomposable organic matters, but also degrades and transforms the cellulose macromolecular substances into soluble small-molecule organic matters that can be utilized by other microbes, and the microbes rapidly multiplied, so that the high temperature period is quickly entered, facilitating the degradation of cellulose, and raising the degradation rate of cellulose (Figure 4C). 15 d later, with the decomposition and consumption of easily decomposable organic matters, the microbes began to degrade the substances hard to decompose, so the composting temperature dropped, and the cellulose degradation rate slightly increased and remained roughly stable. The degradation rate of lignin and cellulose and hemicellulose was increased by 18.1–66.7% compared with CK, and there was a significant difference at value of *p* 0.05 according to multiple comparisons (Figure 4D).

#### 3.2.3. Changes in straw nutrients and humus

The content of active organic matters, N, P, and K in MC rose with the increase of accumulation time in the processes of composting and accumulation. The active organic matters had the highest activity, followed by K and P. At 45 d of accumulation, the content of N, P, and K in the material was 1.51, 0.34 and 0.99%, respectively. At 45 d of natural composting, the content of N, P and K in the material was 0.98, 0.24 and 0.72%, respectively. According to statistical analysis, after manure composting, the content of N, P, and K of bio-composting was significantly higher than that of CK (Figure 2A). Correspondingly, different treatment C/N ratio dropped from 37.25 to 21.40%, 21.92% throughout the process. These phenomena were common and desirable.

The content of fulvic acid (FA) and humic acid (HA) in the combined forms of humus during composting are shown in Figure 2B. After composting, the HA content was 74.06 ± 5.18 g/kg in MC and 49.74 ± 2.29 g/kg in CK, and the FA content was 90.63 ± 7.58 g/kg in MC and 69.56 ± 2.73 g/kg in CK. The most likely reason might be the polymerization of monomolecular FA biodegraded into one part or converted into a complex molecule of another part (such as HA). Generally, a sharp decrease in FA content during composting probably induces the decline in humus (HA + FA) content. On the contrary, the HA content rose by 48.91%, possibly because the organic compounds were rapidly decomposed to produce HA during high-efficiency composting. The HA/FA ratio in MC was increased by 94.02% compared with CK is shown in Figure 2B.

#### 3.2.4. Identification of strains

**Figure 5:**
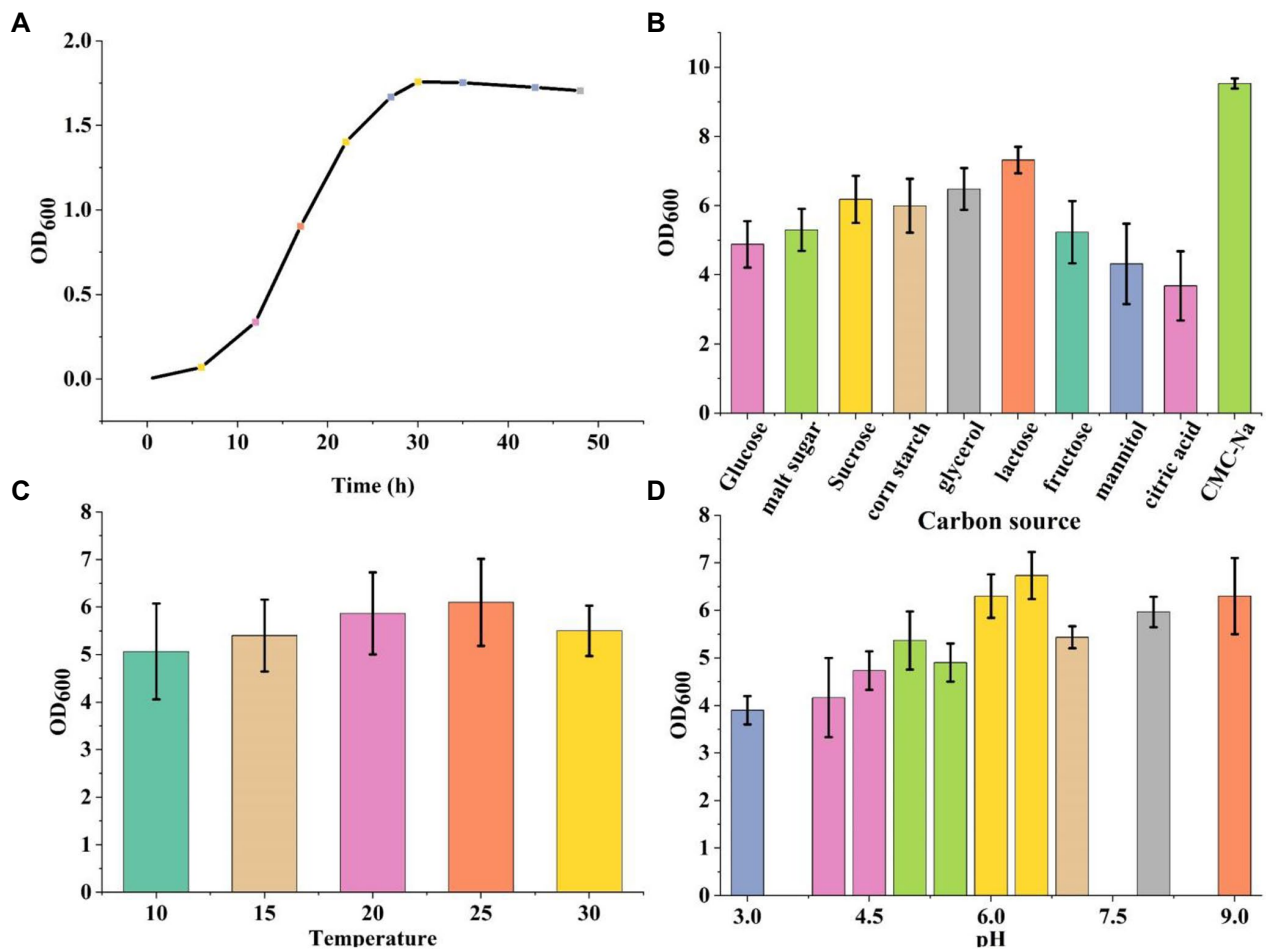
**(A)** The growth curve of *Bacillus subtilis* K1. **(B)** The carbon source of *B. subtilis* K1. **(C)** Temperature of *B. subtilis* K1. **(D)** The pH of *B. subtilis* K1. Vertical bars on graphs indicate the standard deviation of the mean (*n* = 3). Tuckey’s HSD test was used to recognize a significant difference at *p ≤* 0.05 between the treatments.

**Figure 6:**
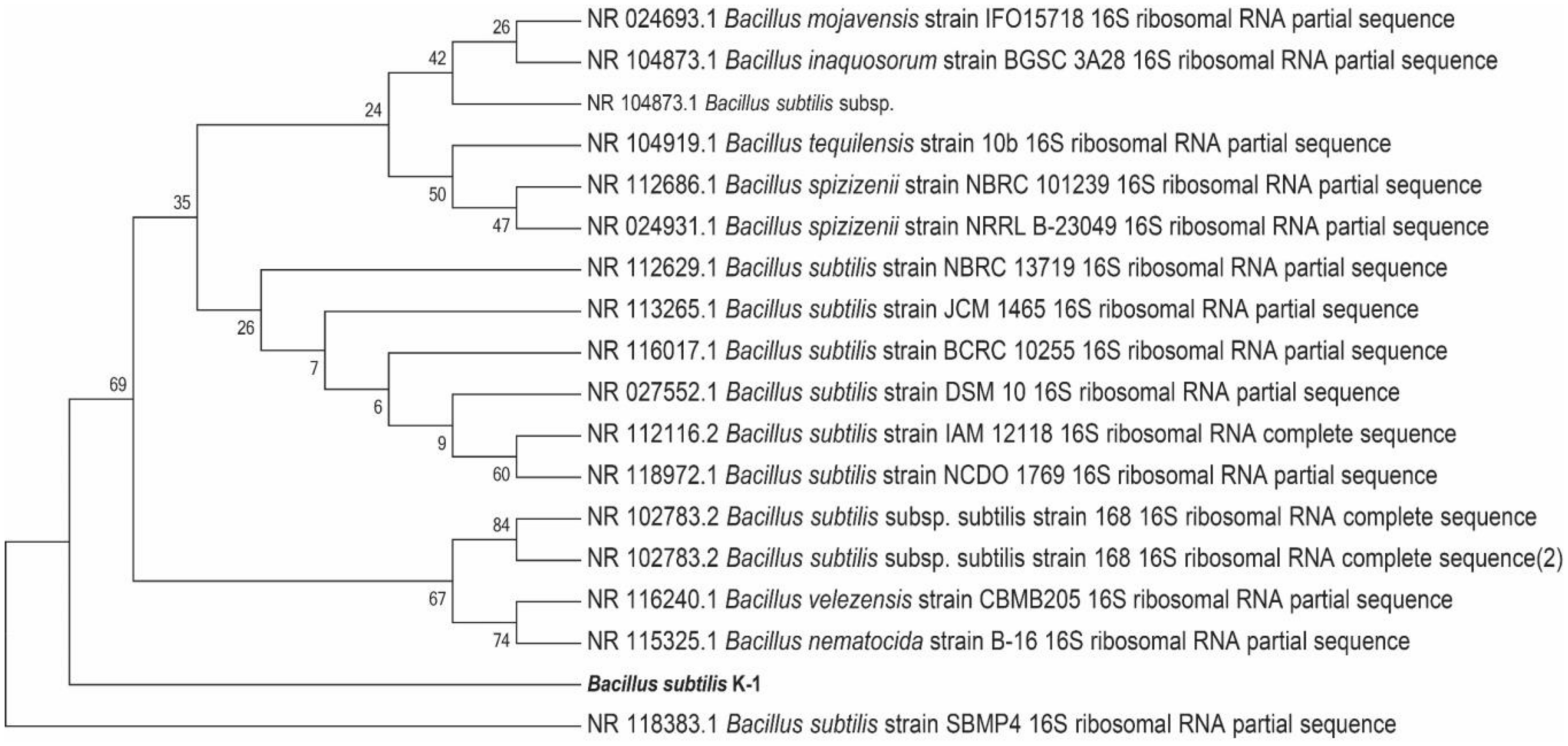
Neighbour-joining tree showing the phylogenetic position of strain *Bacillus subtilis* K1 based on the 16S rDNA gene sequences.

The 16S rRNA gene amplification (using 27F and 1492R primers) and sequencing were conducted for identification of K1. The 16S rRNA gene sequence of K1 was 1,500 bp in length (GenBank ID: SUB12287217), which was submitted to NCBI for BLAST sequence analysis, and a phylogenetic tree was constructed (Figure 5). The results revealed that K1 shared the highest similarity (100%) with *Bacillus subtilis* (NR118383.1), suggesting the highest affinity between K1 and *B. subtilis* (Figure 6).

### 3.3. Genome sequencing of *Bacillus subtilis* K1

#### 3.3.1. Genome sequencing and assembly of *B. subtilis* K1

**Figure 7:**
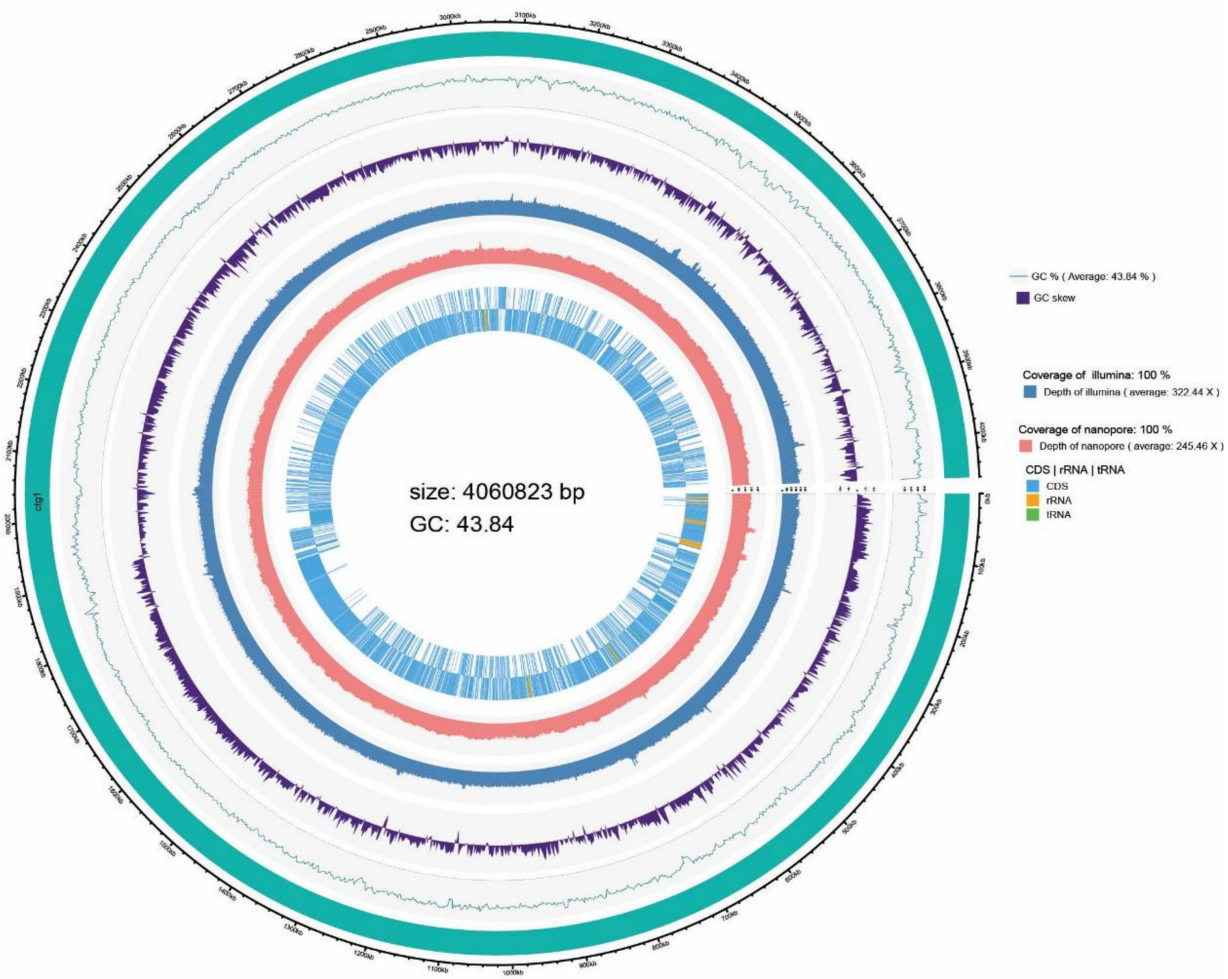
Graphical Circular diagram of the *Bacillus subtilis* K1.

Whole genome sequencing was conducted on *B. subtilis* K1 by the third-generation Pacbio Sequl and the second-generation Illumina Novaseq platform. As shown in Table 1, *B. subtilis* K1 was 4,060,823 bp in genome size, containing 4,213 genes, 10 5S rRNA, 10 16S rRNA and 10 23S rRNA, with 3,665, 3,656, 2,755, 3,240, 1,261, 3,336 and 4,003 genes annotated in the NR, Pfam, COGs, GO, KEGG and Annotation databases, respectively. Furthermore, the genome circle graph of *B. subtilis* K1 was drawn through TBtools software based on the genome-related information (Figure 7).

**Table 1:** Genome statistics for *Bacillus subtilis* K1 strain.

Characteristic	Chromosome
Genome size	4,060,823
GC content	43.44
Gene dosage	4,213
Total gene length	3,641,835
23S rRNA	10
16S rRNA	10
5S rRNA	10
Misc_rna	100
Annotation	4,003
Uniprot	3,865
Pfam	3,656
Refseq	3,965
Nr	3,665
Tigrfam	2,414
GO	3,240
KEGG	1,261
COG	2,755

#### 3.3.2. Clusters of orthologous groups involved in glucose metabolism in genome annotation

**Figure 8:**
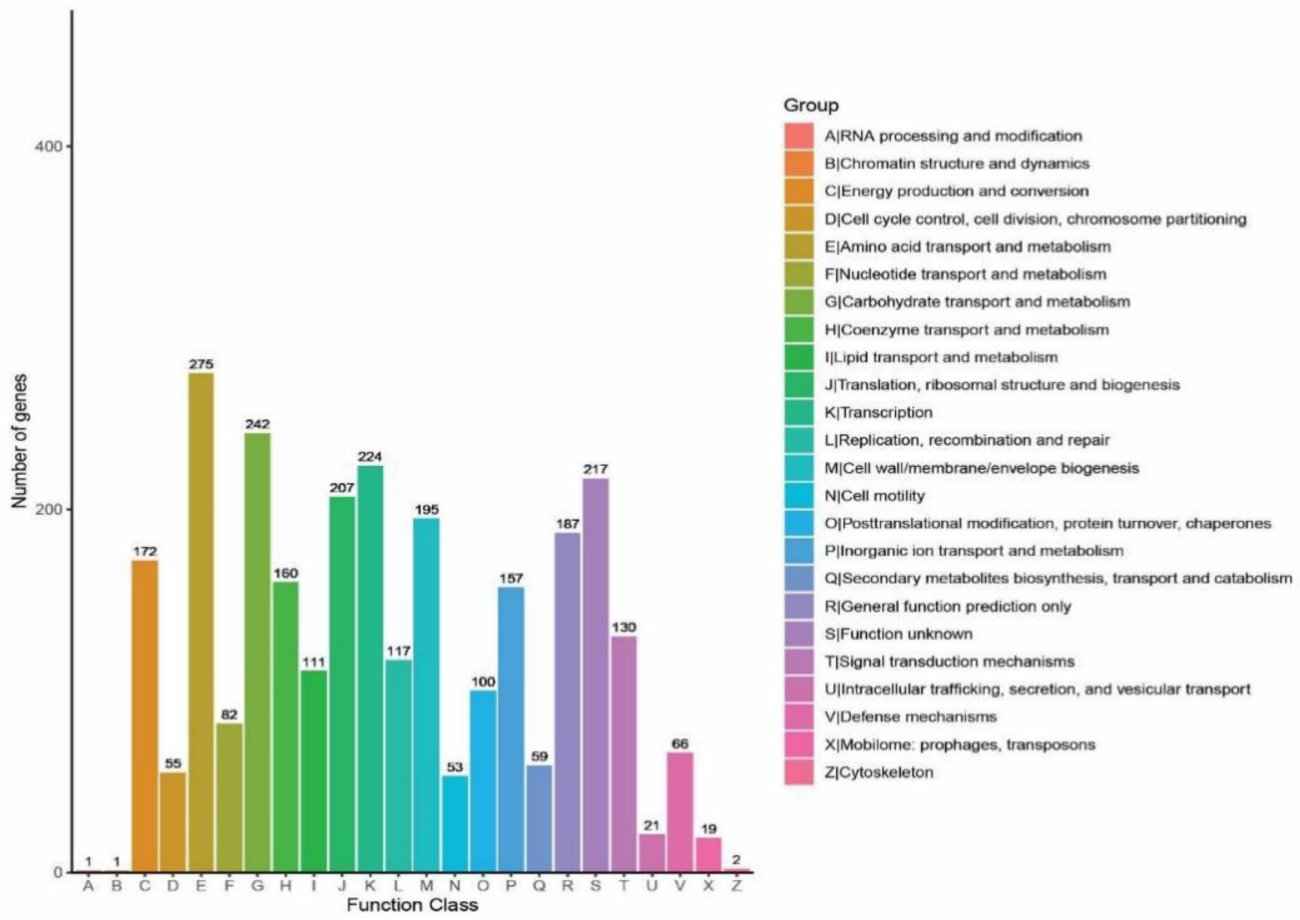
The statistical results of the Clusters of Orthologous Groups (COG) functional classification of genomic protein and analysis of *Bacillus subtilis* K1.

Total 2,775 genes were annotated in COGs, dominated by carbohydrate transport and metabolism (8.82%), amino acid transport and metabolism (9.91%) and transcription (8.07%) (Figure 8). To clarify the potential of *B. subtilis* K1 in cellulose degradation at the genetic level, COGs involved in carbohydrate metabolism were mainly analyzed. A total of 242 genes were annotated in carbohydrate metabolism, in which COG0524 (PfkB domain protein), C and COG2723 (BG) were the most abundant COGs. The high diversity of functional annotations suggested that *B. subtilis* K1 possesses the genetic basis for cellulose degradation.

#### 3.3.3. Go and KEGG annotations

**Figure 9:**
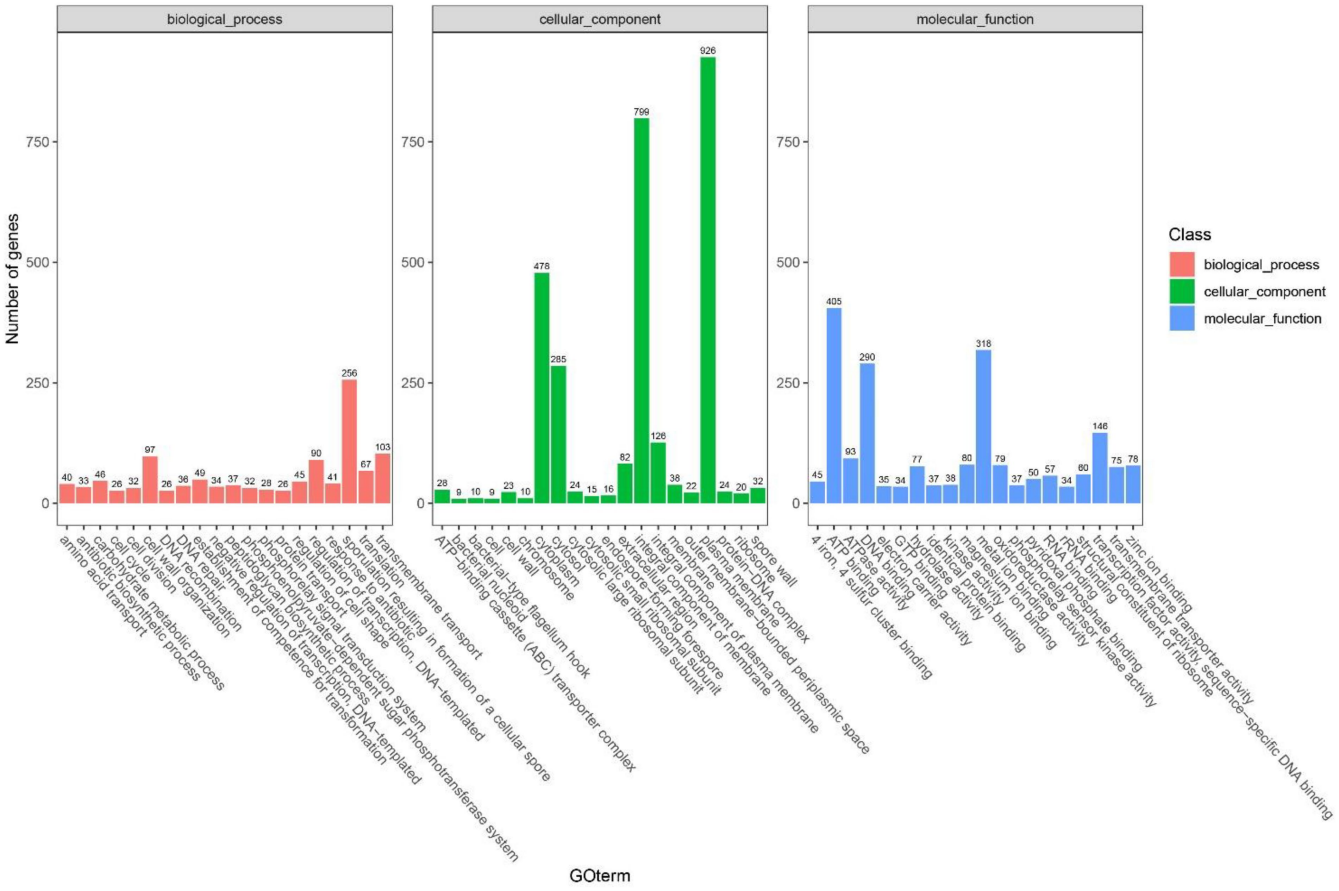
The statistical results of the gene ontology (GO) cluster analysis of *Bacillus subtilis* K1.

It was indicated in GO annotations that genes were mainly enriched in cellular components, followed by molecular functions and biological processes. In terms of cellular components, plasma membrane (926 genes), integral component of membrane (726 genes) and cytoplasm (478 genes) were the most important pathways (Figure 9). With respect to molecular functions, DNA binding (290 genes), ATP binding (405 genes), and metal ion binding (318 genes) were the top 3 important pathways. In addition, 114 GO terms related to carbohydrate metabolism were identified through analyzing GOs related to carbohydrate metabolism, including GO: 0004553 (hydrolase activity that hydrolyzes O-glycosyl compounds), GO:0005975 (carbohydrate metabolism process), and GO:0016787 (hydrolase activity). Among KEGG pathways, metabolism-related pathways had the largest number of genes, followed by environmental information processing. Carbohydrate metabolism in KEGG annotations contained 413 genes, including starch and sucrose metabolism, glycolysis/gluconeogenesis, and pyruvate metabolism (Figure 9).

#### 3.3.4. CAZyme annotations

**Figure 10:**
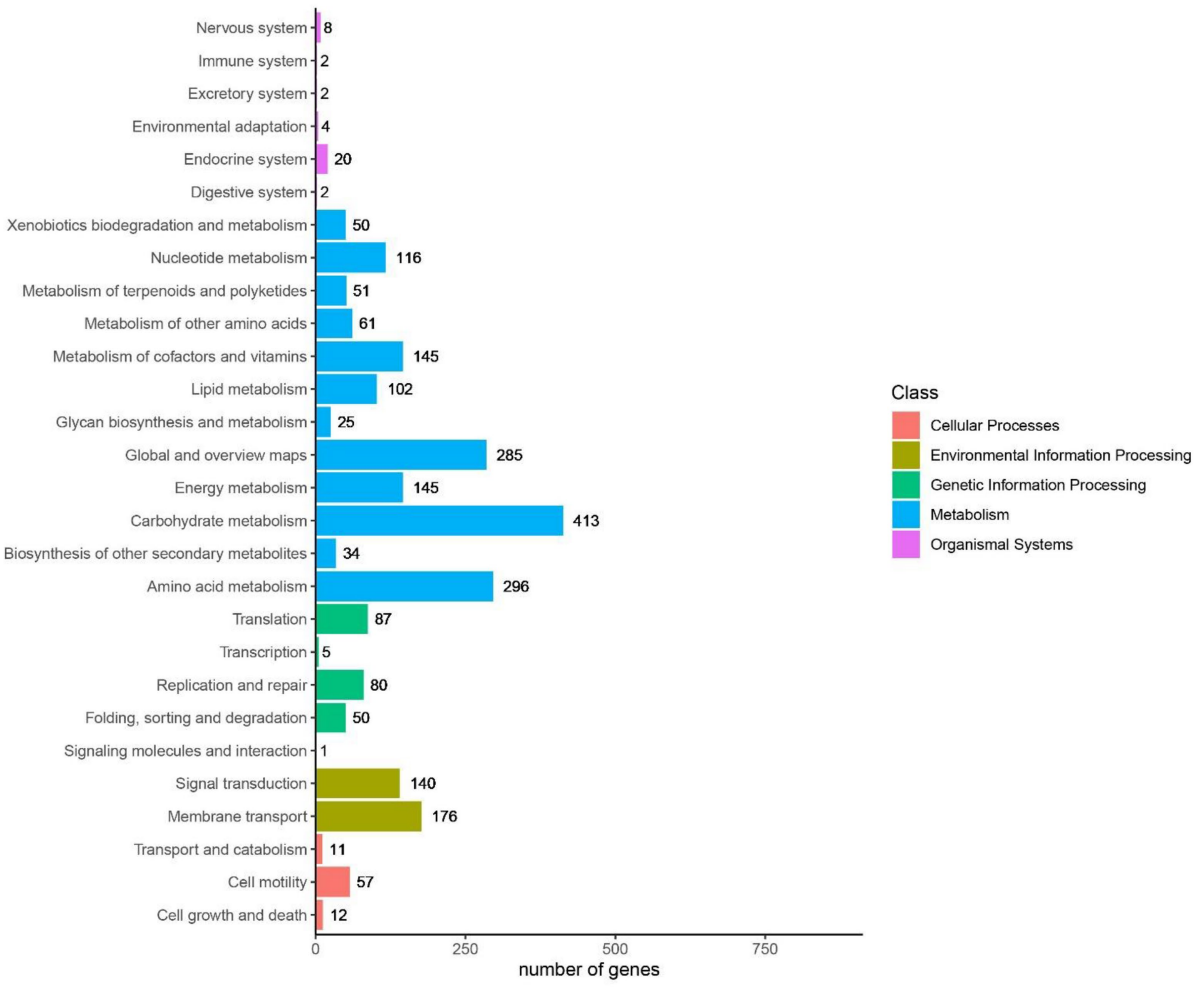
The top Kyoto Encyclopedia of Genes and Genome (KEGG) pathways analysis of *Bacillus subtilis* K1.

CAZyme annotations showed that 125 CAZyme genes were annotated in *B. subtilis* K1 genome, accounting for 2.99% of all coding genes. Among them, glycoside hydrolase (GH) was crucial for the degradation of carbohydrates, and 47 GH genes were annotated in the genome (Figure 10). Besides, 40 glycosyltransferase (GT) genes, 15 carbohydrate esterase (CE) genes, 7 polysaccharide lyase (PL) genes, and 4 enzyme genes with accessory activity (AA) were identified.

#### 3.3.5. Cellulases genes

Total 34 genes related to cellulose degradation were annotated in *B. subtilis* K1 genome, including 13 endoglucanase genes, 10 BG genes, 2 6-phospho-beta-glucosidase genes, 9 α-glucosidase genes, and 1 sucrose-6-phosphate hydrolase gene. The proteins encoded by the endoglucanase genes were members of the GH8 and GH9 families, those encoded by the BG genes were members of the GH1 and GH3 families, those encoded by the α-glucosidase genes mainly belonged to the GH13 and GH18 families, and those encoded by the 6-phospho-beta-glucosidase genes belonged to the GH4 family (Table 2). The abundant cellulose genes suggested that *B. subtilis* K1 possesses strong potential for cellulose degradation.

**Table 2:** Number of genes associated with the *Bacillus subtilis* K1 functional categories.

GENE	Family HMM	Query ID
(Glycoside Hydrolases,GHs)	GH1.hmm	assembly_00394,00642, 04034
	GH3.hmm	assembly_00217
	GH4.hmm	assembly_00782
	GH13_31.hmm	assembly_00332, 03194, 03529
	GH13_28.hmm	assembly_00353
	GH13_29.hmm	assembly_00843
	GH13_14.hmm	assembly_03029
	GH13_9.hmm	assembly_03134
	GH13_20.hmm	assembly_03535
	GH18.hmm	assembly_00024, 03474, 01497, 00628
	GH23.hmm	assembly_01236
	GH26.hmm	assembly_00646, 02660
	GH42.hmm	assembly_03485, 00777
	GH43_11.hmm	assembly_01894
	GH43_16.hmm	assembly_01961
	GH43_5.hmm	assembly_02913
	GH43_4.hmm	assembly_04041
	GH51.hmm	assembly_02880, 02904
	GH53.hmm	assembly_03484
	GH65.hmm	assembly_03530
	GH68.hmm	assembly_03517
	GH73.hmm	assembly_03670, 03176
	GH105.hmm	assembly_03047, 00769
	GH126.hmm	assembly_00485
(Glycosyl Transferases,GTs)	GT1.hmm	assembly_00629, 01318, 02113
	GT2_Glyco_tranf_2_5.hmm	assembly_01882
	GT2_Glyco_tranf_2_3.hmm	assembly_00488
	GT2_Glycos_transf_2.hmm	assembly_00799, 00916, 01391, 01451, 01884, 01885, 02154, 03499, 03501, 03504, 03643, 03657, 03659, 03892, 03899
	GT4.hmm	assembly_03667, 03647, 03505, 03503, 03127, 03124, 02502, 02243
	GT8.hmm	assembly_03945
	GT26.hmm	assembly_03667
	GT28.hmm	assembly_01646, 02184
	GT35.hmm	assembly_03130
	GT46.hmm	assembly_00798
	GT51.hmm	assembly_03849, 03499, 02228
	GT83.hmm	assembly_01390
(Polysaccharide Lyases, PLs)	PL1_6.hmm	assembly_00820
	PL1_8.hmm	assembly_02017
	PL3_1.hmm	assembly_03569
	PL9_2.hmm	assembly_03744
	PL11.hmm	assembly_00774, 00775
	PL26.hmm	assembly_00778
(Carbohydrate Esterases, CEs)	CE1.hmm	assembly_00211, 01267, 03269
	CE4.hmm	assembly_00190, 00860, 01306, 01800
	CE7.hmm	assembly_00367
	CE9.hmm	assembly_03575
	CE10.hmm	assembly_03291, 03511
	CE12.hmm	assembly_04022, 00776
	CE14.hmm	assembly_02118, 02244
(Auxiliary Activities, AAs)	AA7.hmm	assembly_00957, 03524
	AA6.hmm	assembly_00981
	AA4.hmm	assembly_02899
(Carbohydrate-Binding Modules, CBMs)	CBM26.hmm	assembly_00353
	CBM3.hmm	assembly_01958
	CBM6.hmm	assembly_01961
	CBM63.hmm	assembly_02015
	CBM66.hmm	assembly_02717

In *B. subtilis* K1 genome, 9 genes in total encoded hemicellulose, including 3 xylan 1,4-beta-xylosidase genes, 2 beta-galactosidase genes, and 1 arabinogalactan endo-1,4-beta-galactosidase gene, by which the proteins encoded belonged to the GH43 family, GH23 and GH4 families, and GH53 family, respectively. In addition, strain K1 genome contained 9 lignin degradation-related genes, including 2 laccase genes, 1 manganese peroxidase gene, 1 FAD-binding protein gene, and 2 cellobiose dehydrogenase (EC 1.1.99.18) genes (Table 2). The proteins encoded by the laccase gene, manganese peroxidase gene, and cellobiose dehydrogenase gene were members of the AA1 family, AA2 family, and AA3 family, respectively. These results indicate that the strain K1 is able to degrade lignin.

## 4. Discussion

### 4.1. Isolation and identification of cold-adapted cellulose-degrading bacteria

Degradation of crop straw is a bottleneck in natural agricultural settings, there has been a recent increase in the exploration of cold-adapted microorganism, as it can solve the problem of difficult degradation of corn straws under low temperature offering new alternatives for the sustainable development of agriculture. In this study, a cold-adapted cellulose-degrading strain, *B. subtilis* K1, enrichment of culture by cold domestication was utilized. The cellulose activity of *B. subtilis* K1 was higher, among which β-glucanase (24.69 U/ml), exo-β-glucanase (1.72 U/ml) and β-glucosaccharase (1.14 U/ml) were found to have the highest degradation activity. *B. subtilis* K1 is a regular single cell, 2–5.5 μm long and ~ 0.3–0.4 μm wide, with flagellum around its body as shown in its SEM micrograph. In addition, it is a small, round, moist, smooth, viscous, easy to pick, having G+ rod, milk-white round colony with the growing conditions of 5–40°C (optimal temperature 25°C), pH 3.3–8.5 (optimal pH 6.5), optimal carbon source of sodium CMC-Na. It entered the logarithmic period at 8 h, with the highest OD_600_ value at 30 h, and then entered the stabilization period. Despite a strong cellulose-degrading ability, fungi grow more slowly than bacteria, and their cellulase has poor alkali resistance (Ruijssenaars and Hartmans, 2004). Smith et al. (2018) found that *Sphingobacteria* and *Flavobacteria* were the major microbes in different environments of Dry Valley of Antarctica (Huang et al., 2021), proving that such genera are capable of adapting to the low temperature. Cho et al. (2021) isolated Geobacter from the Antarctic soil (Smith et al., 2018), and found that Geobacter is still able to degrade arabinose and hemicellulose in the Antarctic environment. Shang et al. (2012) screened a strain of low temperature-resistant cellulose-degrading bacterium from compost (Cho et al., 2021). The above results demonstrate that *B. subtilis* K1 is expected to be developed into commercially available cellulose biodegrading microbial agents for the sake of comprehensive utilization of straws in Northeastern China.

### 4.2. Changes in physicochemical properties of straw composting

Composting is effective in degrading organic matters, during which carbohydrate metabolism can produce various compounds and degrade lignin and hemicellulose and cellulose under aerobic conditions. As lignin and cellulose and hemicellulose are degraded, the microbes preferentially degrade available degradable substances (Zhang et al., 2016). It can be speculated that carbohydrate metabolism is crucial for the degradation of hemicellulose and cellulose in the process of corn straw composting. In the present experiment, the physicochemical parameters of the cold-adapted cellulose-degrading strain *B. subtilis* K1 applied to the process of low-temperature corn straw composting were different. Specifically, under an average outside temperature of 6°C, after the cold-adapted corn straw decomposing bacteria were added. It was found that through adding cold-adapted cellulose-degrading *B. subtilis* K1 in the corn straw composting under 6°C (ambient temperature), the average temperature of straw composting was 58.7°C higher 86.7% than the control. The HA/FA was higher 94.02% than the control and the lignocellulose degradation rate was lower 18.01–41.39% than the control. Correspondingly, different treatment C/N ratio dropped from 37.25 to 21.40%、21.92% throughout the process. Given the low temperature in Northeastern China, the microbial diversity is high in the straw incorporated soil and the rotten straws, and many types of microbes may degrade the main parts of plants and release nutrients into the environment, thereby participating in nutrient cycling (Li and Jin, 2011). Therefore, it is of great significance to effectively develop a catalytic pathway for microbial degradation of cellulose and prepare an efficient low-temperature straw decomposing microbial agent for solving the problem of straw burning and protecting the black soil in Northeastern China (Yao et al., 2009).

### 4.3. Genome sequencing of *Bacillus subtilis* K1

Whole genome sequencing is an effective means for studying bacterial genetic and molecular mechanisms. In this experiment, a cold-adapted cellulose-degrading strain, *B. subtilis* K1, was screened and subjected to bio-informatics analysis. The results showed that the strain K1 was 4,060,823 bp in genome size and composed of 4,213 genes, among which 3,665, 3,656, 2,755, 3,240, 1,261, 3,336 and 4,003 genes were annotated in the NR, Pfam, COG, GO, KEGG and Annotation databases, respectively, and a large number of lignocellulose degradation-related genes were annotated in the genome. Furthermore, the strain K1 had genes encoding important enzymes (such as GH, GT, PL, CE, and auxiliary oxidoreductase) in the cellulose degradation pathway, and these genes were different in the expression, resulting in different expressions of CMCase, C1, and BG in the strain. These three enzymes act synergistically to completely hydrolyze cellulose GHS family cellulases function as endocellulases and/or exocellulases and play a key role in the early stage of cellulose degradation (Merlin et al., 2014).

## 5. Conclusion

The low-temperature (10°C) and high-efficiency straw-degrading bacterium *B. subtilis* K1 possessed 4,060,823 bp genome size, containing 4,213 genes, with 3,665, 3,656, 2,755, 3,240, 1,261, 3,336 and 4,003 genes annotated in the NR, Pfam, COG, GO, KEGG and Annotation databases, respectively, and large quantities of lignocellulose degradation-related genes annotated in the genome. The cellulose activity of *B. subtilis* K1 was higher, among which β-glucanase (24.69 U/ml), exo-β-glucanase (1.72 U/ml) and β-glucosaccharase (1.14 U/ml) showed highest activity. It was found through the winter corn straw composting experiment that under an average outside temperature of 6°C, after the cold-adapted corn straw decomposing bacteria were added, the average temperature of straw composting (58.7°C) was 86.7% higher than that of the control. The HA/FA ratio in humus was increased by 94.02%, and the degradation rate of lignocellulose was lower 18.01–41.39% than the control. The study results provide a theoretical basis for clarifying the degradation mechanism of cold-adapted straw-degrading bacteria and improving the cellulose degradation efficiency and have instructive significance for the development of high-efficiency straw-degrading bacteria.

## Data availability statement

The datasets generated for this study can be found in NCBI public database. All sequences of 16S rRNA genes and whole genome sequence can be found in Sequence Read Archive (SRA) under Bio Sample accession no. PRJNA902081.

## Author contributions

YJ and ZH planned and designed the research. ZH performed research and contributed to the methodology, writing, and editing. HL, YZ, and XW assisted in analysis, and compiled the data and results. HD and YH helped in experiments and improved the manuscript. QA, BD, and YJ critically revised the manuscript. All authors contributed to the article and approved the submitted version.

## Funding

This work was funded by the LiaoNing Revitalization Talents Program (Project no. XLYC1807221) and Shenyang City Science and Technology Plan (Project no. 21–109–3-01).

## Conflict of interest

PD was employed by General Manager, Contec Global Agro Ltd., Nigeria.

The remaining authors declare that the research was conducted in the absence of any commercial or financial relationships that could be construed as a potential conflict of interest.

## Publisher’s note

All claims expressed in this article are solely those of the authors and do not necessarily represent those of their affiliated organizations, or those of the publisher, the editors and the reviewers. Any product that may be evaluated in this article, or claim that may be made by its manufacturer, is not guaranteed or endorsed by the publisher.
